# Diversity of *Mycoplasma genitalium* strains in Australia: relationship with sexual networks and antimicrobial resistance

**DOI:** 10.1007/s10096-025-05081-0

**Published:** 2025-03-03

**Authors:** Teck-Phui Chua, Jennifer A. Danielewski, Emma L. Sweeney, Erica L. Plummer, Catriona S. Bradshaw, David M. Whiley, Dorothy A. Machalek, Suzanne M. Garland, Gerald L. Murray

**Affiliations:** 1 Department of Obstetrics, Gynaecology and Newborn Health, University of Melbourne, Parkville, VIC Australia; 2 Centre for Women’s Infectious Diseases, The Royal Women’s Hospital, Parkville, VIC Australia; 3 Molecular Microbiology Research Group, Murdoch Children’s Research Institute, Parkville, VIC Australia; 4 Centre for Clinical Research, The University of Queensland, Brisbane, QLD Australia; 5 School of Translational Medicine, Monash University, Melbourne, VIC Australia; 6 Melbourne Sexual Health Centre, Alfred Health, Melbourne, VIC Australia; 7 Melbourne School of Population and Global Health, University of Melbourne, Parkville, VIC Australia; 8 Pathology Queensland Central Laboratory, Brisbane, QLD Australia; 9 The Kirby Institute, University of New South Wales, Sydney, NSW Australia

**Keywords:** Mycoplasma genitalium, Antibiotic resistance, Macrolide, Fluoroquinolone, *MgpB*, Molecular typing

## Abstract

**Purpose:**

Molecular typing can identify relationships between *M. genitalium* strains and antimicrobial resistance and demographic data. We examined the association of *mgpB* sequence types (STs) with sex/sexual orientation, antimicrobial resistance and geographical location for *M. genitalium* in Australia.

**Methods:**

Sequence data derived from previous studies in Victoria and Queensland were obtained from 170 *M. genitalium* samples for the *mgpB*, 23 S rRNA, and *parC* genes. An additional 55 *M. genitalium* samples from Victoria were sequenced for the same three genes in this study. A combined data set of 225 samples collected between 2017 and 2019 were examined for associations between *mgpB* ST and (i) sex/sexual orientation, (ii) macrolide and fluoroquinolone resistance, and (iii) geographical location using chi-square test.

**Results:**

Overall, 66 *mgpB* STs were identified; the most common were ST-7 (17.9%), ST-4 (11.6%), ST-105 (11.6%), and ST-2 (5.4%). There was a strong association between ST and sex/sexual orientation; ST-4 and ST-105 were most common among men-who-have-sex-with-men (*p* < 0.0001) while ST-7 among women (*p* < 0.0001). There was a strong association between ST and macrolide resistance (*p* = 0.0028). Fluoroquinolone resistance was less common (28.0%) and did not differ by STs (*p* = 0.20). There was no association between ST and geographic location (*p* = 0.056).

**Conclusion:**

In this Australian study, four *mgpB* STs were common and were strongly associated with sex/sexual orientation and macrolide resistance. This relationship was not seen for fluoroquinolone resistance nor geographic location. These findings highlight the sporadic nature of resistance, indicating a need for effective treatment approaches combined with routine antimicrobial resistance surveillance.

**Supplementary Information:**

The online version contains supplementary material available at 10.1007/s10096-025-05081-0.

## Introduction

*Mycoplasma genitalium* is a sexually transmitted bacterium of significant public health concern due to increasing levels of antimicrobial resistance [1]. Resistance to the two common antibiotics used for treatment, azithromycin and moxifloxacin, have increased over the past years, especially in the Western Pacific where levels reached 47% and 41% in 2018–21, respectively [2]. Levels of infection and antimicrobial resistance are higher in men-who-have-sex-with-men (MSM) compared to other groups such as men-who-have-sex-with-women (MSW) and women [2]. Of concern is the risk of antimicrobial resistance spread from MSM networks to other sexual networks [3].

Two typing methods are commonly used for *M. genitalium* [4], one targeting the *mgpB* (MG191) adhesin gene [5] and the other the MG309 lipoprotein gene [6]. The *mgpB* typing system involves the sequencing of a 281 bp non-repeat region of the *mgpB* gene. Unlike other regions of this gene, this non-repeat region does not undergo recombination but has sufficient sequence variation to distinguish between strains [7]. The MG309 lipoprotein gene has a highly variable number of short tandem repeats (STRs) consisting of a various number of two trinucleotide repeat units, AGT and AAT [6] and has also been used for typing [8–15]. Other genes have been evaluated for typing but have demonstrated relatively lower discriminatory power (< 0.8730) compared to the *mgpB* and MG309 genes (0.9334 to 0.9634 individually, and 0.9894 combined), indicating a lesser ability to distinguish between strains [8, 16]. The combination of two or more loci for typing achieves greater discriminatory power compared to using a single locus. While higher resolution is important for studies analysing strains within a patient, or for studies examining transmission networks, single locus-based typing is sufficient to facilitate general epidemiological analyses [9, 17].

Several studies have examined the relationship of strains with resistance, sex, and sexual orientation [3, 8–12, 18]. While no clear association has been identified, studies from Europe have observed that *mgpB* sequence type (ST) 4 is more commonly found among MSM [9, 12, 13, 17]. Whether this is a global phenomenon and its impact on the spread of antimicrobial resistance is unknown. Previous typing studies from Australia have had a narrow geographic focus. The aim of this study was to use single locus-based typing to examine the association between *mgpB* STs and geographical location, antimicrobial resistance, sex, and sexual orientation in Australia.

## Materials and methods

This study examines a total of 225 samples: a combination of previously acquired sequence data (from 170 *M. genitalium* samples), and sequencing data generated in this study (from 55 *M. genitalium* samples), as described below.

### *Mycoplasma genitalium* sequence data from previous studies

Sequence data for the *mgpB*, 23 S rRNA and *parC* genes from 170 *M. genitalium*-positive clinical samples collected from patients between 2017 and 2019 were obtained from four studies [10, 19–21]. In brief, these samples were from two Australian states; 89 samples from Queensland [10], 81 samples from Victoria (29 samples from [21] and 52 samples from [19, 20]. Sequencing of the *mgpB* gene was performed using either Sanger sequencing [10, 20], or on the Illumina platform [21]. Macrolide resistance was defined as 23 S rRNA gene mutations A2071G/C/T and A2072G/C (*M. genitalium* numbering) and was determined either using the SpeeDx ResistancePlus MG assay [10, 19], or Illumina sequencing [21]. The genotype of the *parC* gene was determined using Sanger sequencing [10, 20] or Illumina sequencing [21], whereby fluoroquinolone resistance was defined as mutations in the *parC* gene corresponding to amino acid changes S83I/R/N and D87Y/N/H (*M. genitalium* numbering) [22, 23].

Ethical approval was granted by The Children’s Health Queensland Human Research Ethics Committee (HREC/12/QRCH/139) and the Alfred Hospital Human Research Ethics Committee (278/16 and 490/16).

### Sequencing of *M. genitalium* positive clinical samples

*M. genitalium* positive samples (n = 55) obtained from a previous study [24] that investigated the contribution of *M. genitalium* to urogenital symptoms in women were sequenced in this study. These samples were collected from women attending the Melbourne Sexual Health Centre (MSHC) between 2017–2019. In this study, the samples were sequenced for the *mgpB* [25], 23S rRNA [21] and *parC* [22] genes using previously described primers with Oxford Nanopore tails at the 5’ end of the primer (forward tail sequence: TTTCTGTTGGTGCTGATATTGC and reverse tail sequence: ACTTGCCTGTCGCTCTATCTTC). Each 15 µl PCR reaction consisted of 1X Platinum™ SuperFi™ PCR Master Mix (Invitrogen), 0.25 µM each of forward and reverse primers, nuclease-free water and DNA. Cycling conditions were as follows: initial denaturation at 98ºC for 30 s, 35 cycles of 98ºC for 10 s, 60ºC for 10 s, and 72ºC for 15 s, and final extension at 72ºC for 5 min.

Amplicons were prepared for sequencing using a ligation sequencing kit with a barcoding expansion pack (SQK-LSK114 with EXP-PBC096) and sequenced using the FLOW-MIN114 R10.4.1 flow cell on the MinION (Oxford Nanopore Technologies, Oxford, UK). Reads were basecalled with the super accurate protocol using Dorado version 0.3.0 (https://github.com/nanoporetech/dorado) before demultiplexing and adaptor and barcode trimming with Guppy version 6.4.6. Variant calling was performed using Geneious Prime version 2023.2.1 (Biomatters Ltd, Auckland, New Zealand). Ethical approval was granted by the Alfred Hospital Ethics Committee (100/17) and all patients provided written informed consent.

### Sequence typing analysis

The *mgpB* sequences from *M. genitalium* samples were aligned to the *M. genitalium* type strain G37 (GenBank accession number NC_000908.2) with MUSCLE [26] using Geneious Prime version 2023.2.1 (Biomatters Ltd, Auckland, New Zealand). Minimum spanning trees were generated by the goeBURST algorithm using PHYLOViZ 2.0 [27, 28]. Naming of *mgpB* STs can be found in Supplementary Table 1. The SNP distance between *mgpB* STs was determined using the tool snp-dists (https://github.com/tseemann/snp-dists) on Galaxy version 0.8.2 + galaxy0.

### Statistical analysis

Demographic data included location (Queensland or Victoria, Australia), sex/sexual orientation (MSM, men-who-have-sex-with-men-and-women [MSMW], MSW, or women [W]), and specimen type (anorectal, urine, or cervicovaginal). Anorectal samples from men without corresponding sexual orientation data were categorised as MSM (*n* = 13). Associations between demographic factors and antibiotic resistance markers with commonly detected STs were examined by chi-square test using GraphPad version 9.1.1 (GraphPad Software, Massachusetts, USA).

## Results

### Sequencing results and sample characteristics

Of the 55 *M. genitalium* samples sequenced in this study, *mgpB* sequences were successfully obtained for 53 samples; the remaining samples contained a mixed infection and the predominant *mgpB* strain could not be elucidated. For macrolide and fluoroquinolone resistance, sequences for the 23 S rRNA gene were available for all 55 samples, and sequences for the *parC* gene were available for 42 samples. Novel *mgpB* sequences identified were deposited in PubMLST for *mgpB* assignment (ST-331 to ST-338) and GenBank (accession numbers: PQ424956–PQ424967); these came from women in Victoria.

These sequences were combined with those from previous studies published in Australia resulting in a total of 225 *M. genitalium* samples, 89 (39.6%) from Queensland (population of 5 million), and 136 (60.4%) from Victoria (population of 6.4 million). The majority of samples were from women (34.2%) and MSM (30.2%) (Table 1).

**Table 1:** Sample characteristics of the 225 *Mycoplasma genitalium* samples collected between 2017–2019 analysed in this study

	All samples	Sweeney et al. [10]	Plummer et al. [21]	Read et al. [19] & Chua et al. [20]	This study with samples from [24]
**Number of samples**	225	89	29	52	55
**Location**					
Queensland	89 (39.6%)	89 (100%)	0 (0%)	0 (0%)	0 (0%)
Victoria	136 (60.4%)	0 (0%)	29 (100%)	52 (100%)	55 (100%)
**Sex/sexual orientation**					
MSM	68 (30.2%)	16 (18.0%)	0 (0%)	52 (100%)	0 (0%)
MSMW	1 (0.44%)	1 (1.1%)	0 (0%)	0 (0%)	0 (0%)
MSW	38 (16.9%)	38 (42.7%)	0 (0%)	0 (0%)	0 (0%)
W	77 (34.2%)	22 (24.7%)	0 (0%)	0 (0%)	55 (100%)
Unknown	41 (18.2%)	12 (13.5%)	29 (100%)	0 (0%)	0 (0%)
**Specimen type**					
Anorectal	58 (25.8%)	19 (21.3%)	0 (0%)	34 (65.4%)	0 (0%)
Cervicovaginal	68 (30.2%)	17 (19.1%)	0 (0%)	0 (0%)	49 (89.1%)
Urine	66 (29.3%)	40 (44.9%)	0 (0%)	18 (34.6%)	0 (0%)
Unknown	33 (14.7%)	13 (14.6%)	29 (100%)	0 (0%)	6 (10.9%)
**Macrolide resistance mutations** ^**a**^					
Yes	153 (68%)	51 (57.3%)	26 (89.7%)	48 (92.3%)	28 (50.9%)
No	71 (31.6%)	38 (42.7%)	2 (6.9%)	4 (7.7%)	27 (49.1%)
Unknown	1 (0.44%)	0 (0.0%)	1 (3.4%)	0 (0%)	0 (0%)
**Fluoroquinolone resistance mutations** ^**b**^					
Yes	63 (28%)	50 (56.2%)	6 (20.7%)	6 (11.5%)	1 (1.8%)
No	144 (64%)	39 (43.8%)	19 (65.5%)	45 (86.5%)	41 (74.5%)
Unknown	18 (8.0%)	0 (0.0%)	4 (13.8%)	1 (1.9%)	13 (23.6%)
**Dual class resistance mutations** ^**c**^					
Yes	50 (22.2%)	37 (41.6%)	6 (20.7%)	6 (11.5%)	1 (1.8%)
No	157 (69.8%)	52 (58.4%)	19 (65.5%)	45 (86.5%)	41 (74.5%)
Unknown	18 (8.0%)	0 (0.0%)	4 (13.8%)	1 (1.9%)	13 (23.6%)

MSM: men-who-have-sex-with-men; MSMW: men-who-have-sex-with-men-and-women; MSW: men-who-have-sex-with-women; W: women

^**a**^ Macrolide resistance defined as a single nucleotide polymorphism at position 2058/2059 of the 23 S rRNA or a positive result as determined by the SpeeDx ResistancePlus assay

^**b**^ Fluoroquinolone resistance defined as an amino acid change in ParC S83 or D87

^**c**^ Samples with both macrolide and fluoroquinolone resistance. Unknown samples were either unsuccessfully sequenced for the 23 S rRNA gene, the *parC* gene, or both

### Clustering of *mgpB* types

**Fig. 1:**
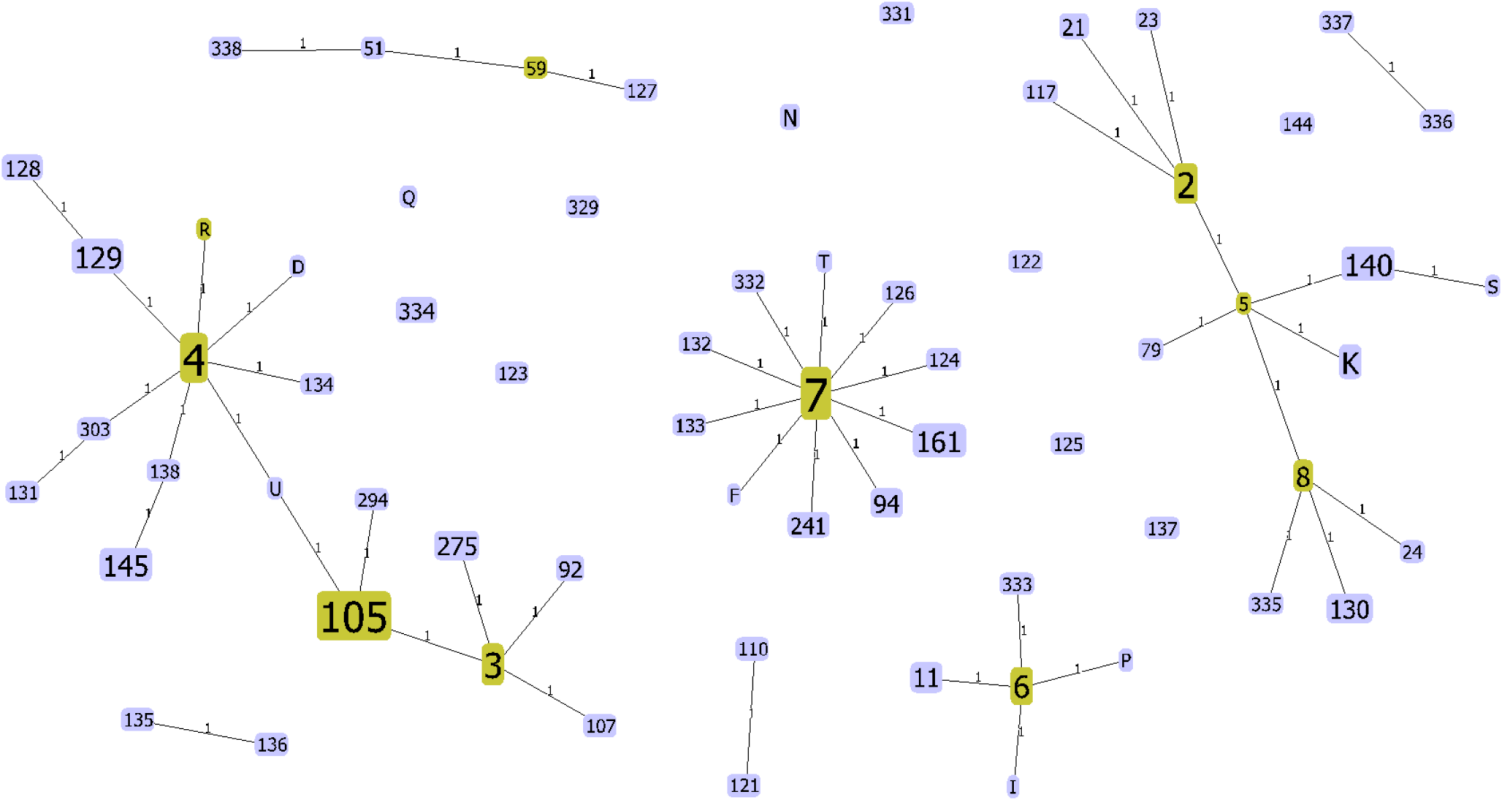
Minimum spanning tree based on *mgpB* sequences. The number in each node represents the *mgpB* sequence type (ST). Yellow nodes represent the group founder as determined by the goeBURST algorithm. Blue nodes are common nodes. Node size is proportional to the number of samples. Lines indicate one SNP difference between two nodes and are not drawn to scale. Clonal complexes are defined by more than one single nucleotide difference. The main clonal complexes are around ST-4/105, ST-7 and ST2

Out of the 225 samples, 223 had a corresponding sequence for the *mgpB* gene. A total of 66 *mgpB* STs were identified in our study, with SNP differences ranging from one to 18 SNPs. The Simpson’s diversity index was 0.9325, and the most common STs were ST-7 (17.9%), ST-4 (11.6%), ST-105 (11.6%), and ST-2 (5.4%; Table 2). In clonal complex analysis, the main clusters formed around these common STs, and ST-6 (Fig. 1). Interestingly, ST-4 and ST-105 were closely related, clustering together in the same clonal complex with a difference of two SNPs (Fig. 1).

**Table 2:** Demographic and antimicrobial resistance data for the four main *MgpB* sequence types identified

	All *mgpB* sequences	ST-4	ST-105	ST-7	ST-2	p-value
**Number of samples**	223	26	26	40	12	
**Sex/sexual orientation**						
MSM	68	15 (57.7%)	19 (73.1%)	2 (5.0%)	0 (0.0%)	< 0.0001^a^
MSMW	1	0 (0.0%)	0 (0.0%)	0 (0.0%)	0 (0.0%)	
MSW	38	4 (15.4%)	1 (3.8%)	5 (12.5%)	4 (33.3%)	
W	75	6 (23.1%)	0 (0.0%)	25 (62.5%)	4 (33.3%)	
Unknown	41	1 (3.8%)	6 (23.1%)	8 (20.0%)	4 (33.3%)	
**Specimen type**						
Anorectal	58	13 (50.0%)	13 (50.0%)	2 (5.0%)	0 (0.0%)	< 0.0001^b^
Cervicovaginal	66	5 (19.2%)	0 (0.0%)	22 (55.0%)	3 (25.0%)	
Urine	66	7 (26.9%)	8 (30.8%)	6 (15.0%)	6 (50.0%)	
Unknown	33	1 (3.8%)	5 (19.2%)	10 (25.0%)	3 (25.0%)	
**Macrolide resistance** ^**c**^						
Yes	152	20 (76.9%)	24 (92.3%)	23 (57.5%)	5 (41.7%)	0.0028
No	70	6 (23.1%)	2 (7.7%)	17 (42.5%)	7 (58.3%)	
Unknown	1	0 (0.0%)	0 (0.0%)	0 (0.0%)	0 (0.0%)	
**Fluoroquinolone resistance** ^**d**^						
Yes	63	4 (15.4%)	7 (26.9%)	12 (30.0%)	3 (25.0%)	0.20
No	144	22 (84.6%)	19 (73.1%)	17 (42.5%)	9 (75.0%)	
Unknown	16	0 (0.0%)	0 (0.0%)	11 (27.5%)	0 (0.0%)	
**Dual class resistance** ^**e**^						
Yes	50	4 (15.4%)	7 (26.9%)	7 (17.5%)	1 (8.3%)	0.49
No	157	22 (84.6%)	19 (73.1%)	22 (55.0%)	11 (91.7%)	
Unknown	16	0 (0.0%)	0 (0.0%)	11 (27.5%)	0 (0.0%)	
**Geographic location**						
Queensland	89	6 (23.1%)	8 (30.8%)	12 (30.0%)	8 (66.7%)	0.056
Victoria	134	20 (76.9%)	18 (69.2%)	28 (70.0%)	4 (33.3%)	

MSM: men-who-have-sex-with-men; MSMW: men-who-have-sex-with-men-and-women; MSW: men-who-have-sex-with-women; W: women

^**a**^ MSM vs. non-MSM (MSW & W) and W vs. non-W (MSM & MSW). P-value was 0.037 for MSW vs. non-MSW (MSM & W)

^**b**^ Note all except one anorectal sample were provided by MSM (one was from MSMW) and all cervicovaginal samples were provided by women

^c^Macrolide resistance defined as a single nucleotide polymorphism at position 2058/2059 of the 23 S rRNA or a positive result as determined by the SpeeDx ResistancePlus assay

^**d**^ Fluoroquinolone resistance defined as an amino acid change in ParC S83 or D87

^**e**^ Samples with both macrolide and fluoroquinolone resistance. Unknown samples were either unsuccessfully sequenced for the 23 S rRNA gene, the *parC* gene, or both

### Association of *mgpB* sequence types with sex and sexual orientation

The factors associated with the four most commonly detected STs were further explored (Supplementary Fig. 1). A significant association was identified between ST and sex/sexual orientation (Table 2). The majority of ST-4 and ST-105 samples were from MSM (*p* < 0.0001), while ST-7 was most common in women (*p* < 0.0001) and ST-2 was mostly associated with both women and MSW (*p* = 0.037). When considering anatomical site of collection there was also an association with *mgpB* ST, although it is important to note that these associations are confounded by the fact that MSM provided most of the anorectal samples and women provided the cervicovaginal samples (Table 2). *M. genitalium* in anorectal samples were mostly ST-4 and ST-105, cervicovaginal samples mostly ST-7 and urine samples were consistently reported in all four common STs.

### Association of *mgpB* sequence types with resistance mutations

**Fig. 2:**
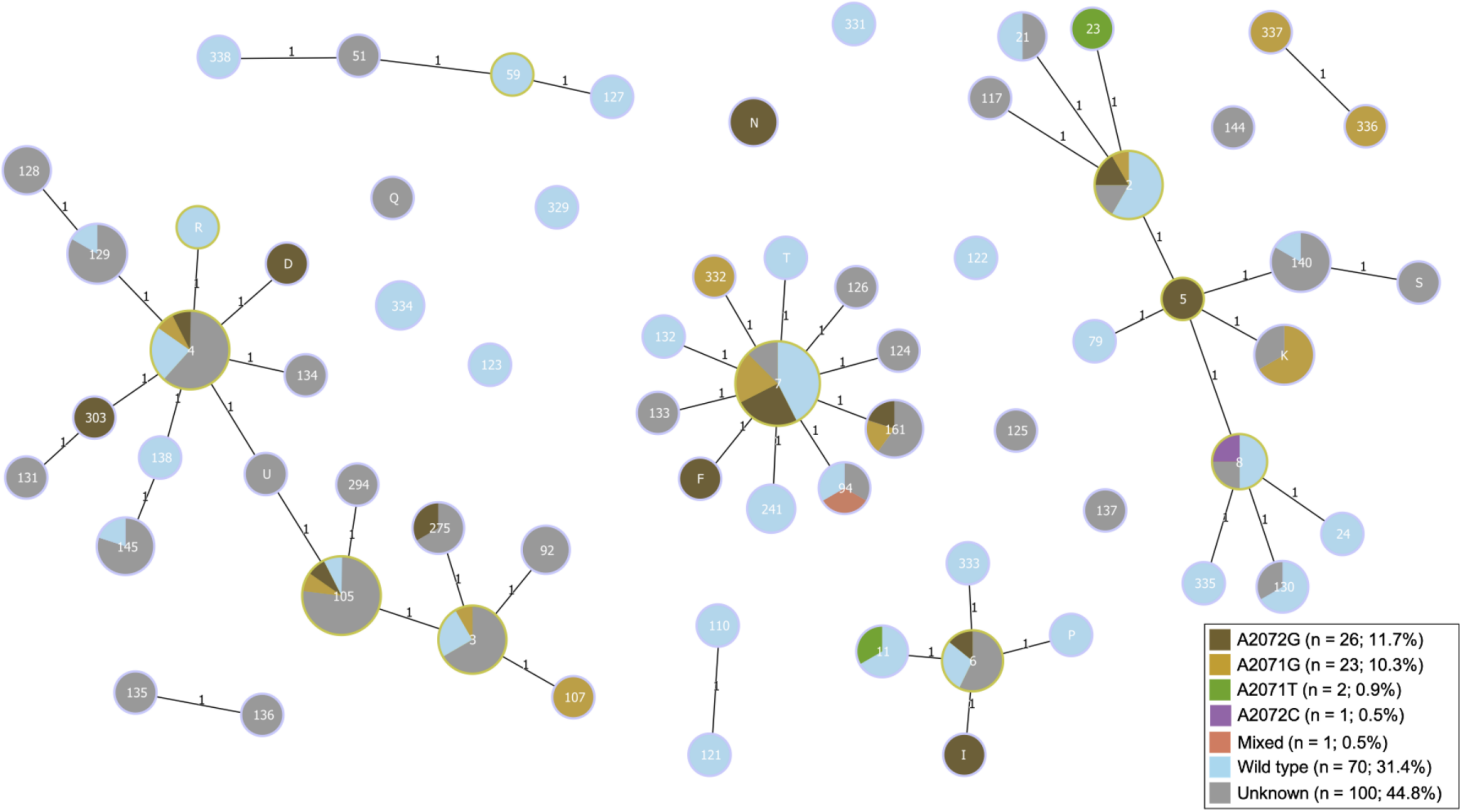
Distribution of single nucleotide polymorphisms (SNPs) at positions A2071 and A2071 of the 23 S rRNA, conferring macrolide resistance, among sequence types. The number in each node represents the *mgpB* sequence type (ST). Node size is proportional to the number of samples. Lines indicate one SNP difference between two nodes and are not drawn to scale. Samples with an unknown 23 S rRNA genotype were either due to unsuccessful sequencing or due to the use of the SpeeDx ResistancePlus MG assay which does not distinguish between 23 S rRNA SNPs

Macrolide resistance mutations were found in 68.0% of samples and were distributed across STs. Of note, a higher proportion of samples belonging to ST-4 and ST-105 (76.9% and 92.3%, respectively) harboured macrolide resistance mutations compared to ST-7 and ST-2 (57.5% and 41.7%, respectively; *p* = 0.0028; Table 2). From available data in two studies [21, 24], analysis of individual 23 S rRNA mutations conferring macrolide resistance showed that the most common single nucleotide polymorphisms were A2072G (11.7%) and A2071G (10.3%). There was a variation of 23 S rRNA mutations among STs (Fig. 2). Fluoroquinolone resistance mutations were present in 28.0% of samples but was not associated with specific *mgpB* STs (*p* = 0.20; Table 2). STs were also not associated with geographic location (*p* = 0.056; Table 2).

## Discussion

We identified 66 *mgpB* STs in our study of 225 samples collected in Queensland and Victoria between 2017 and 2019. The most common STs were ST-4, ST-105, ST-7, and ST-2. Analysis of clonal complexes identified clustering around these same STs with ST-4 and ST-105 clustered together with a difference of two SNPs. There was a strong association between ST and sex/ sexual orientation, and macrolide resistance, however, no clear relationship between ST and fluoroquinolone resistance nor geographical location was observed.

The finding of dominant STs bears similarity to other recent studies. In Spain, samples from 2014 to 2018 contained the same four predominant *mgpB* STs (9.5% ST-4, 11.4% ST-105, 13.3% ST-7 and 10.5% ST-2) [8]. These STs were also identified in South Africa between 2015 and 2019, with ST-7 and ST-2 most prevalent (18.2% and 13.6%, respectively) [11]. Of these, ST-4 has been the most widely reported in studies from France [12, 14], Germany [9, 13, 29], and Spain [3].

Emerging evidence suggests that certain strains are more common in specific populations, indicating spread within a sexual network. Molecular typing analysis by Fernández-Huerta et al. (2020) identified two distinct clusters which were significantly associated with sexual orientation; women and MSW composed 64.7% of one group and MSM and MSMW made up 83.8% of the other group [3]. Similarly, in our study, we observed some *mgpB* STs were more common among MSM (ST-4 and ST-105), while others were more common to MSW and women (ST-7 and ST-2). Several studies have also commonly identified ST-4 among MSM [9, 12, 13, 17, 18] and a study from France observed that ST-2 was predominant in MSW, although, no significant difference in *mgpB* STs was identified between MSM and MSW populations [12].

Alternatively, the prominence of certain strains in specific populations may be a consequence of strain-specific pathogenesis or tissue tropisms. ST-4 and ST-105 were common among anorectal samples from MSM in this study. Similarly, Guiraud et al. (2021) found that ST-4 was highly prevalent in rectal samples from MSM. It has been postulated that ST-4 transmits more easily via the anorectal route, but this, and its association with symptoms of proctitis, remains unknown [12, 13].

Macrolide resistance was common among all STs but was especially high in ST-4 and ST-105 (76.9% and 92.3%, respectively). This is most likely because the majority of samples in ST-4 and ST-105 were from MSM, a group that has nearly double the number of macrolide-resistant *M. genitalium* infections compared to MSW [2]. While macrolide resistance was significantly associated with ST, no ST was exclusively composed of macrolide resistant genotypes, and no STs harboured only one specific 23 S rRNA gene mutation. This suggests that clonal transmission of resistant strains in Australia is unlikely and supports previous studies in other countries which observed that the rise in macrolide resistance was due to *de novo* acquisition of 23 S rRNA gene mutations rather than the spread of a resistant strain [8, 30–34].

In contrast, fluoroquinolone resistance was not associated with ST, however, fluoroquinolone resistance was not observed as frequently as macrolide resistance in our dataset which consisted of samples collected between 2017 and 2019. A recent study found a predominance of dual ParC and GyrA mutations among ST-130 and ST-146, suggestive of clonal spread [15]. However, neither of these two STs were common in our study, and we did not investigate GyrA mutations as they were rarely observed in our dataset [10, 20, 21]. Nevertheless, other studies suggest polyclonal spread of fluoroquinolone resistance in *M. genitalium*, resulting from the independent acquisition of mutations during treatment [3, 9, 10, 12].

There are limitations to our study. First, a single gene was used for typing which may be too discriminatory, however, this method is sufficient for the purposes of our study and facilitates comparisons with findings from other studies which commonly use the *mgpB* gene. Currently there is no established multi-locus typing system for *M. genitalium*; whole genome sequencing studies would be useful to reinforce the utility of *mgpB* as a typing tool and may identify new markers to develop a multi-locus sequence typing scheme for *M. genitalium*. Second, data were only available from only two states in Australia, Victoria and Queensland. However, these two states represent over 40% of the national population, providing a good understanding of circulating strains within our population. Third, samples were collected from 2017 to 2019, therefore, results may not reflect the current state of *M. genitalium* in Australia, especially those for fluoroquinolone resistance which are expected to be higher in recently collected samples. Fourth, the genotypes of the 23 S rRNA gene were only available for samples from two studies [21, 24] consisting mostly of women, so our results may not be generalisable to MSM. Finally, due to our defined population, several factors may have confounded any associations present such as unequal distribution of MSM samples in each state, although, our observations support those from separate analyses of the two datasets performed previously [10, 20]. A larger sample size may assist with finding associations between *mgpB* ST and investigated factors.

In conclusion, there was a strong association between *mgpB* STs and sex/sexual orientation, specimen type, and macrolide resistance. We found that the associations between ST and sexual orientation reported in Germany and France were also observed in Australia [9, 12, 13, 17]. This association between STs and sexual orientation indicates strong sexual networks within populations which has implications for the spread of resistance; ST-4 and ST-105 were predominant among MSM samples and were associated with macrolide resistance. Despite this, macrolide resistance was not restricted to certain STs, adding to the growing body of evidence that macrolide resistance in *M. genitalium* is widespread and develops independently during treatment. This association also further highlights the need for highly effective treatments, including resistance-guided therapy approaches, particularly for fluoroquinolones as suggested previously [35, 36]. Ultimately, structured surveillance programs to monitor the development and spread of resistance are required as this will inform treatment guidelines and control initiatives.

## Electronic supplementary material

Below is the link to the electronic supplementary material.

Supplementary Material 1

## Data Availability

Novel *mgpB* sequences identified were deposited in PubMLST for *mgpB* assignment (ST-331 to ST-338) and GenBank (accession numbers: PQ424956–PQ424967).
