# Supplementary material for: Diversity of Mycoplasma genitalium strains in Australia: relationship with sexual networks and antimicrobial resistance
Source: Eur J Clin Microbiol Infect Dis. 2025 Mar 3;44(5):1167–75. doi: 10.1007/s10096-025-05081-0 (PMC12062162; doi:10.1007/s10096-025-05081-0)
Supplement: Supplementary file 1 — Supplementary Material 1 [file 10096_2025_5081_MOESM1_ESM.docx]

**Supplementary material**

**Supplementary Table 1.** Naming of *mgpB* sequences identified in this study compared to other naming systems.

| **This study** | **Pineiro *et al.*, 2019 [1]** | **Dumke *et al.*, 2019 [2]** | **Sweeney *et al*., 2020 [3]** | **Plummer *et al.*, 2020 [4]** | **PubMLST [5]** |
| --- | --- | --- | --- | --- | --- |
| 2 | 2 |  | 2, 74 | M | 2 |
| 3 | 3 |  | 3, 78 | B | 3 |
| 4 | 4 |  | 4 | C | 4 |
| 5 | 5 |  | 5 |  | 5 |
| 6 | 6 |  | 6 | O | 6 |
| 7 | 7 |  | 7 | E | 7 |
| 8 | 8 |  | 8 | L | 8 |
| 11 | 11 |  | 11, 136 | H | 11 |
| 21 | 21 |  | 21 |  | 21 |
| 23 | 23 |  | 23 | J | 23 |
| 24 | 24 |  | 24 |  | 24 |
| 51 | 51 |  | 51 |  | 51 |
| 59 | 59 |  | 59 |  | 59 |
| 79 | 79 |  | 79 |  | 79 |
| 92 | 92 |  | 92 |  | 92 |
| 94 | 94 |  | 94 |  | 94 |
| 105 | 105 |  | 105 | A | 105 |
| 107 | 107 |  | 107 |  | 107 |
| 110 | 110 |  | 110, 128 |  | 110 |
| 117 | 117 |  | 117 |  | 117 |
| 121 |  |  | U1 |  | 121 |
| 122 |  |  | U10 |  | 122 |
| 123 |  |  | U11 |  | 123 |
| 124 |  |  | U12 |  | 124 |
| 125 |  |  | U13 |  | 125 |
| 126 |  |  | U14 |  | 126 |
| 127 |  |  | U15 |  | 127 |
| 128 |  |  | U16 |  | 128 |
| 129 |  |  | U17 |  | 129 |
| 130 |  |  | U18 |  | 130 |
| 131 |  |  | U2 |  | 303 |
| 132 |  |  | U3 |  | 132 |
| 133 |  |  | U4 |  | 133 |
| 134 |  |  | U5 |  | 134 |
| 135 |  |  | U6 |  | 135 |
| 136 |  |  | U7 |  | 136 |
| 137 |  |  | U8 |  | 137 |
| 138 |  |  | U9 |  | 138 |
| 140 |  | 159 | 159 |  | 140 |
| 144 |  |  |  |  | 144 |
| 145 |  | 130 | 130 |  | 145 |
| 161 |  | 143 | 143 | G | 161 |
| 241 |  | 141 | 141 |  | 241 |
| 275 |  | 127 | 127 |  | 275 |
| 294 |  |  |  |  | 294 |
| 303 |  |  |  |  |  |
| 329 |  |  |  |  |  |
| 331 |  |  |  |  |  |
| 332 |  |  |  |  |  |
| 333 |  |  |  |  |  |
| 334 |  |  |  |  |  |
| 335 |  |  |  |  |  |
| 336 |  |  |  |  |  |
| 337 |  |  |  |  |  |
| 338 |  |  |  |  |  |
| D |  |  |  | D |  |
| I |  |  |  | I |  |
| F |  |  |  | F |  |
| K |  |  |  | K |  |
| N |  |  |  | N |  |
| P |  | 147 | 147 |  |  |
| Q |  |  |  |  |  |
| R |  |  |  |  |  |
| S |  |  |  |  |  |
| T |  |  |  |  |  |
| U |  |  |  |  |  |

**A)**


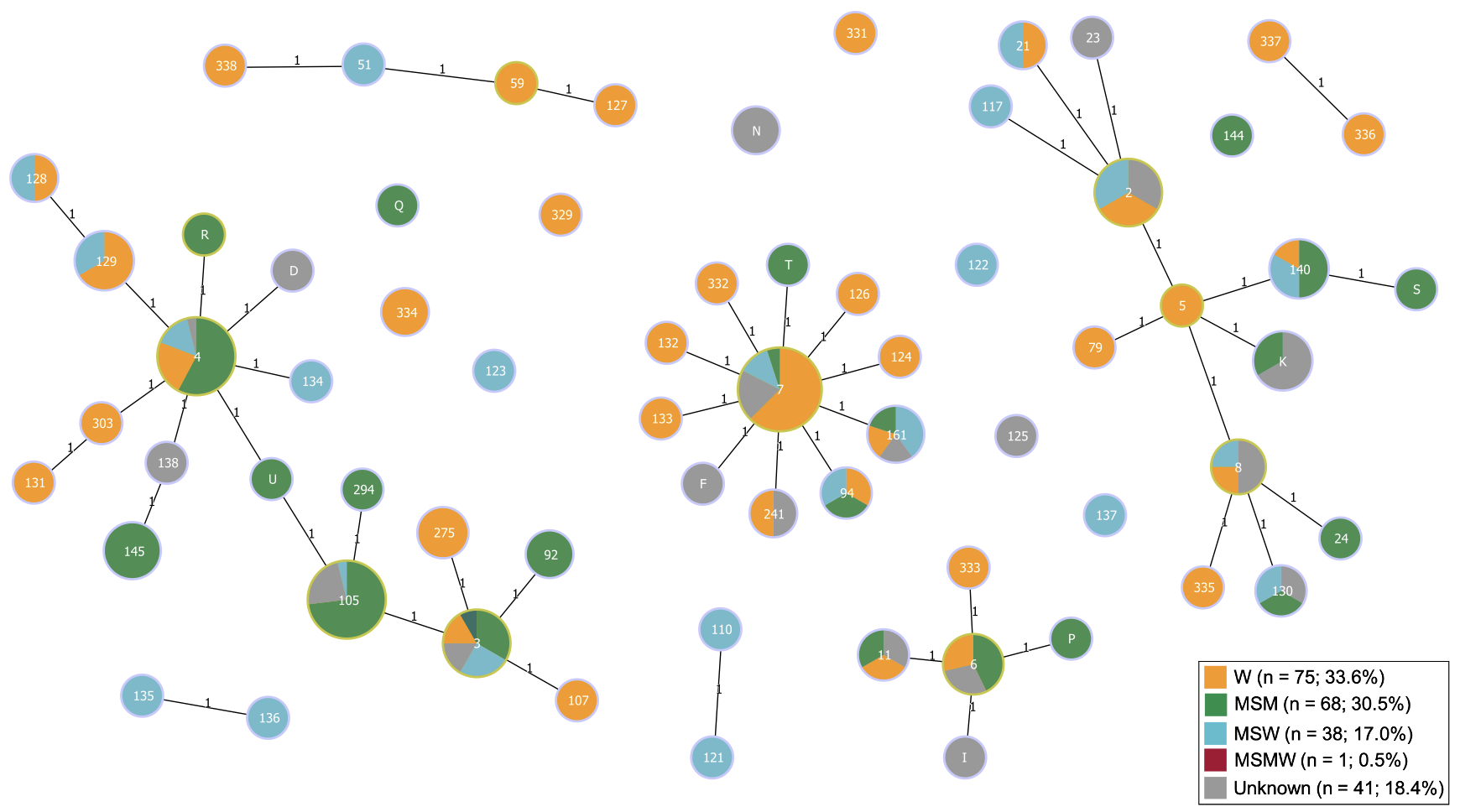


**B)**


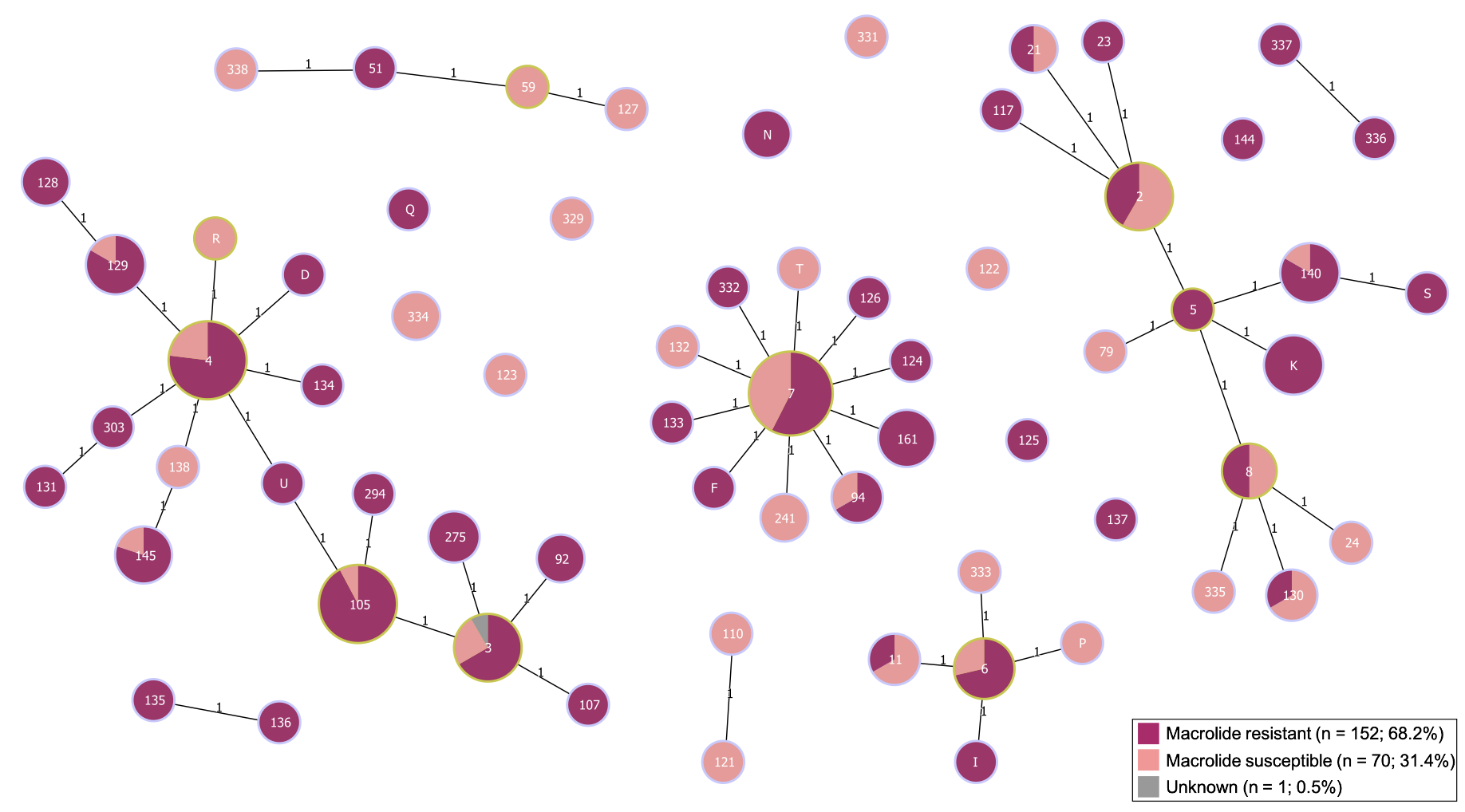


**C)**


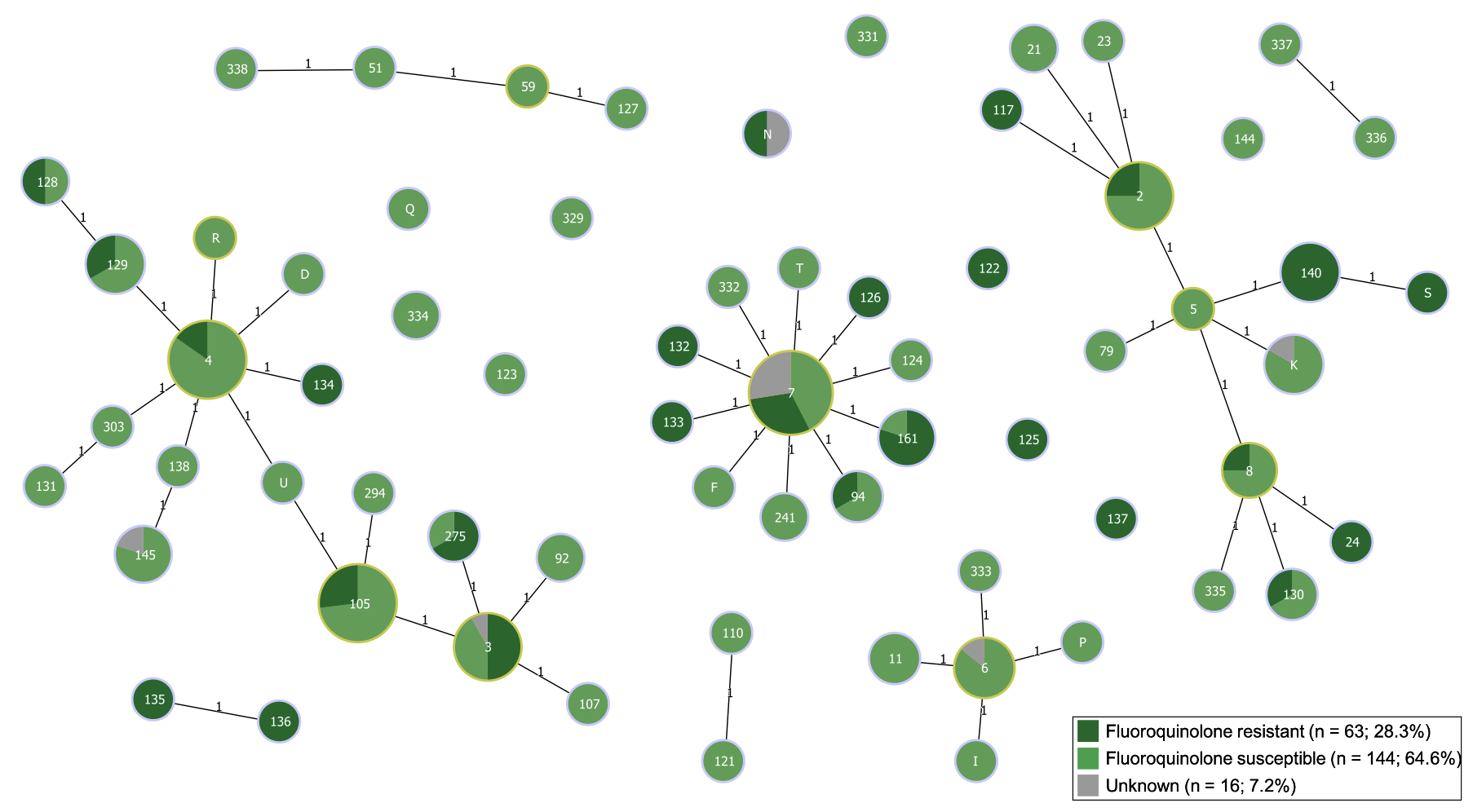


**D)**


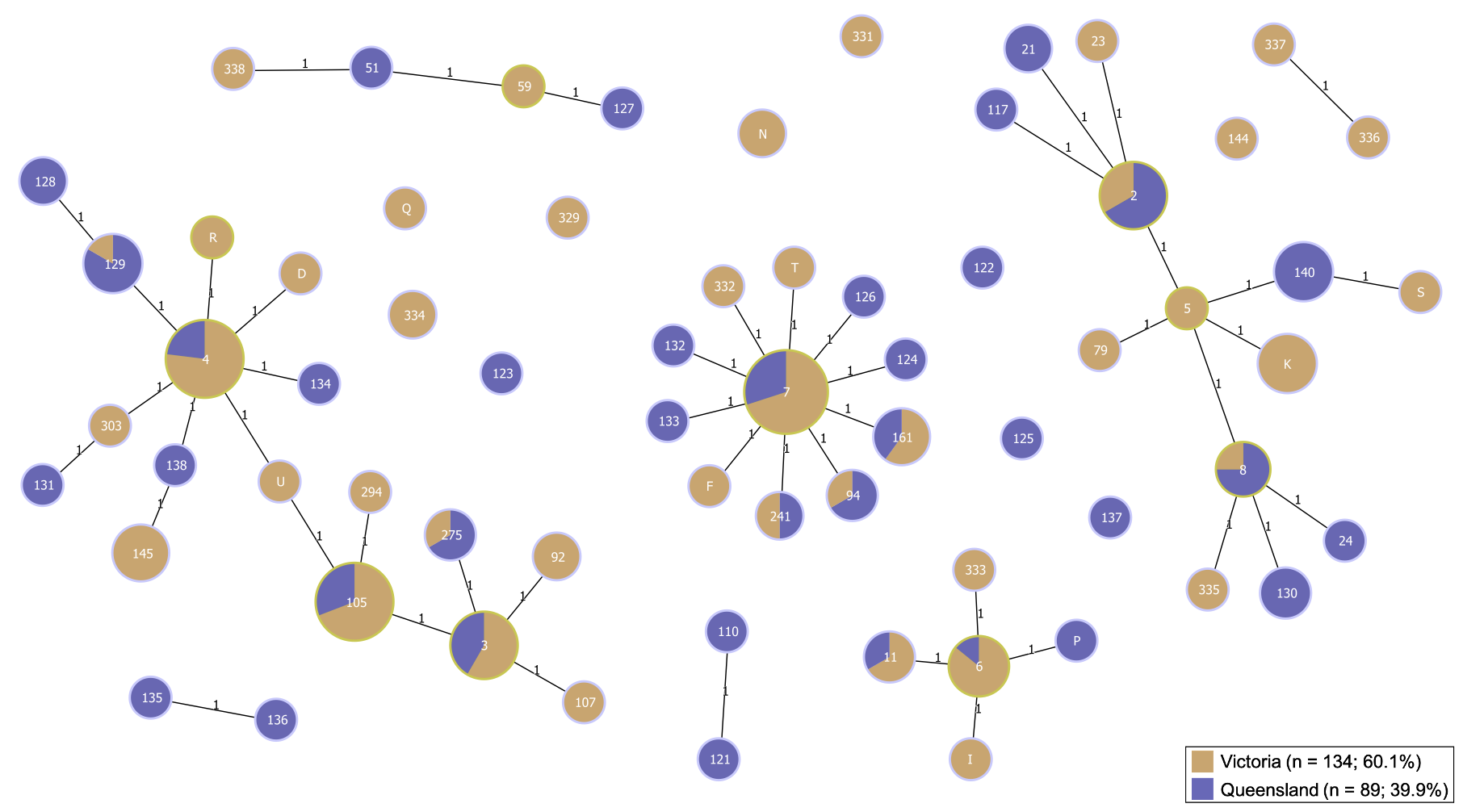


**Supplementary Figure 1.** Minimum spanning trees of *mgpB* sequence types (STs) of 170 *Mycoplasma genitalium* samples from Queensland and Victoria, Australia by sex/sexual orientation (A), macrolide resistance (B), fluoroquinolone resistance (C), and location (D). The size of each circle is proportional to the number of samples. Numbers in each circle represent the *mgpB* ST (Supplementary Table 1). Lines indicate one SNP difference between two nodes and are not drawn to scale. *mgpB* ST was strongly associated with sex/sexual orientation (p < 0.0001) and macrolide resistance (p = 0.0028; Table 2).

1. Piñeiro L, Idigoras P, Cilla G (2019) Molecular typing of *Mycoplasma genitalium*-positive specimens discriminates between persistent and recurrent infections in cases of treatment failure and supports contact tracing. Microorganisms 7 (12):609

2. Dumke R, Rust M, Glaunsinger T (2019) *MgpB* types among *Mycoplasma genitalium* strains from men who have sex with men in Berlin, Germany, 2016-2018. Pathogens 9 (1):12

3. Sweeney EL, Tickner J, Bletchly C, Nimmo GR, Whiley DM (2020) Genotyping of *Mycoplasma genitalium* suggests *de novo* acquisition of antimicrobial resistance, Queensland, Australia. Journal of Clinical Microbiology 58 (9):e00641-00620

4. Plummer EL, Murray GL, Bodiyabadu K, Su J, Garland SM, Bradshaw CS, Read TRH, Tabrizi SN, Danielewski JA (2020) A custom amplicon sequencing approach to detect resistance associated mutations and sequence types in *Mycoplasma genitalium*. Journal of Microbiological Methods 179:106089

5. Jolley KA, Bray JE, Maiden MCJ (2018) Open-access bacterial population genomics: BIGSdb software, the PubMLST.org website and their applications. Wellcome Open Res 3:124
